# Experiences of primary care for people with dementia from socioeconomically disadvantaged areas: a qualitative study

**DOI:** 10.3399/BJGP.2025.0407

**Published:** 2026-03-10

**Authors:** Charlotte Morris, Dame Louise Robinson, Darren M Ashcroft, Evangelos Kontopantelis, Tom Blakeman

**Affiliations:** 1 Division of Population Health, Health Services Research and Primary Care, School of Health Sciences, The University of Manchester, Manchester, UK; 2 NIHR Greater Manchester Patient Safety Research Collaborative, The University of Manchester, Manchester, UK; 3 NIHR School for Primary Care Research, The University of Manchester, Manchester, UK; 4 Population Health Sciences Institute, Faculty of Medical Sciences, Newcastle University, Newcastle-upon-Tyne, UK; 5 Division of Pharmacy & Optometry, School of Health Sciences, The University of Manchester, Manchester, UK; 6 Division of Informatics, Imaging and Data Sciences, The University of Manchester, Manchester, UK

**Keywords:** dementia, health inequality, primary health care, qualitative research, socioeconomic disparity, socioeconomic status

## Abstract

**Background:**

The increasing prevalence of dementia is expanding the role of primary care in its management. However, inequities exist in primary care for people with dementia, particularly related to socioeconomic status. Individuals from socioeconomically disadvantaged areas are underrepresented in research on this topic.

**Aim:**

To explore primary care experiences of people with dementia and their carers from socioeconomically disadvantaged areas.

**Design and setting:**

Qualitative interviews with people with dementia and their carers recruited from deprived localities (Index of Multiple Deprivation quintile 1 or 2 postcodes).

**Method:**

We undertook 20 semi-structured interviews. Participants were purposively sampled by postcode via the Join Dementia Research database. Participants were aged >18 years, with any type of dementia or were a carer for someone with dementia. Data were analysed using reflexive thematic analysis.

**Results:**

We identified four interrelated themes. These were: proactive continuity as a means of maintaining identity amid impending loss; the paradox of healthcare support falling away as it becomes needed more; challenges with accessing and navigating the primary healthcare system; and uncertainty around where dementia health care sits within primary care. Views about the impact of socioeconomic disadvantage varied. Some participants felt local resources, or individual social capital, affected the health care they received.

**Conclusion:**

Changes in primary care, including proactive contact, clearer communication of dementia-related care, and consistent relationships could improve patient experience. Clarifying primary care’s role in dementia management is essential, especially with proposed shifts towards primary care-led post-diagnostic support. Further work is needed to establish if these experiences are described by people with dementia across all social strata or are specific to areas of socioeconomic disadvantage.

## How this fits in

Existing national guidance recommends primary care-led dementia health care, but little is known about the experience of this for people living in socioeconomically deprived areas. This study highlights that people with dementia, and their carers, in socioeconomically disadvantaged areas want to maintain identity and understand their decline. Support from healthcare services often diminishes over time, with difficulties accessing and navigating healthcare systems when needed. There was uncertainty about primary care’s role in dementia health care. Clearer communication and proactive support from primary care may improve experiences for these patients.

## Introduction

The World Health Organization describes dementia as a global public health concern as cases rapidly increase.^1^ In the UK, the number of people diagnosed with dementia is projected to increase from 985 000 currently, to 1.6 million by 2050.^2,3^ Despite the diagnosis of dementia usually being made by a specialist in the UK, most health care for people with dementia is provided by primary care.^4,5^ UK guidance suggests people with symptoms of cognitive impairment should be referred to a memory clinic for assessment and formal diagnosis.^6^ There are inequalities in access to these services.^7^

Increasing numbers of people with dementia means greater involvement of primary care.^3^ The World Alzheimer’s Report 2022^8^ recommends health care for people with dementia should be primary care-led, with associated benefits for patients, families and carers, healthcare professionals, and health systems. These include long-term relationships and co-ordinating care.^8,9^ A task-shifted approach prioritising primary care is increasingly adopted as demand on specialist services increases.^8^ Dementia is complex, requiring ongoing support from health and social care for the person affected and their wider network. Primary care provides social, holistic, not just medical, input for people with dementia, in a person-centred way.

A range of healthcare delivery models have been utilised to provide health care for people with dementia globally, with no single model being used consistently.^10^ A systematic review identified four main models: primary care-led, primary care-led with specialist consulting, case-management partnerships, and integrated models; no single model represented best practice.^11^ Recent evidence suggests a ‘Care Ecosystem’ model, with a trained, telephone-based navigator, collaborating with specialists, provided benefit.^12^ Uncertainty around best practice is evidenced even at regional level; there is varied commissioning of services, with concerns of a ‘postcode lottery’ of what is available where, and for whom.^13^

Qualitative work has explored key components of post-diagnostic support from the perspectives of people with dementia and their carers.^14^ Important healthcare components included maintaining and improving cognition and care planning, and supporting physical health.^14^ Important holistic components included information provision, care coordination, safeguarding, advocacy, and continuity.^14^ Many of these components fall within the remit of primary care and are detailed in healthcare guidance.^6,8^

There is evidence primary care clinicians feel well placed to provide health care for people with dementia,^5,15^ but they lack training, knowledge, and confidence.^5,15–17^ In the UK, the dementia domains of the Quality and Outcomes Framework are to keep an accurate register of people with dementia, and to record a face-to-face review for these people annually. Box 1 details guidance for the annual review.^18,19^ Both domains have high levels of achievement,^19^ meaning clinicians and practices are coding these in health records. This does not necessarily translate to high-quality health care. Qualitative evidence suggests that people with dementia are dissatisfied with primary health care,^20,21^ and may be unaware that care planning/reviews have taken place.^20^

Box 1. **Suggested components of the care plan for people with dementia, which should be reviewed at least annually. Adapted from National Institute for Health and Care Excellence^18^**

- Diagnosis review including the subtype
- Review of carer(s) support
- Medication review
- Risk evaluation
- New symptoms inquiry
- Treatments and support offered
- Advanced care planning
- Individualised information

As the role of primary care in providing health care for people with dementia evolves, understanding the views of people with dementia and their carers about existing primary care provision is important. There is evidence that provision of guideline-consistent primary care for people with dementia varies with socioeconomic factors,^22–24^ therefore it is particularly important to understand the views of those who typically experience health disadvantage, who may experience additional barriers in navigating the primary healthcare system.^25,26^ Despite this, few studies specifically focusing on the views of people with dementia from areas of socioeconomic deprivation about their experiences of primary care were identified in a literature review prior to commencing this study. In this qualitative study we aimed to understand the views of people with dementia and their carers from areas of socioeconomic deprivation and their experiences of primary care.

## Method

### Design and recruitment

The study is reported in line with standard reporting of qualitative research recommendations;^27^ ethical approval was obtained from an NHS Research Ethics Committee (reference: 24/WM/0102). The research design aligned with interpretivism, understanding the subjective meanings and experiences of individuals. We used an inductive analytic approach, building our theories, concepts, and eventual themes from the raw data. We interviewed people with dementia and carers of people with dementia between August 2024 and March 2025. Participants were recruited from the Join Dementia Research (JDR) database, a securely managed database of volunteers interested in participating in dementia research (https://www.joindementiaresearch.nihr.ac.uk). This database includes people with dementia, informal and professional carers, and people without dementia but who act as healthy volunteers (controls).

We purposively sampled people from socioeconomically deprived parts of the UK. To determine areas of greater socioeconomic deprivation we used the Index of Multiple Deprivation (IMD).^28,29^ The IMD is an area-level composite measure of relative deprivation, ranking small areas of the UK on seven different domains indicating relative poverty or affluence.^28^ Anyone living in an IMD 1 or 2 quintile area or from populations who typically experience health disadvantage,^30^ such as minority ethnicity, disability, and/or multimorbidity, was eligible for inclusion. Carers and people with dementia living in their own homes or in care homes were eligible.

We aimed primarily to recruit individuals living in IMD 1 or 2 postcode areas. To achieve this, partial postcodes covering IMD 1 and 2 areas were sent to the JDR database administrators, matching participants full postcodes were then cross-referenced against UK postcodes’ IMD level.^31^ Participants who were determined to be from IMD 1 or 2 quintiles were contacted and asked if they wished to take part. Participants with characteristics that typically result in health disadvantage, such as comorbidities and disability, were eligible if they contacted the study team, but were not proactively recruited. This meant we focused on socioeconomic deprivation without excluding people with relevant experiences just because of current postcode.

Participants or carers were contacted through JDR, depending on who was the named preferred contact. People with dementia were asked if they wanted to also invite a carer to participate with them, and carers were asked if they wanted to invite the person with dementia.

A patient and public involvement and engagement (PPIE) group guided study methodology. They contributed to the interview topic guide, interview techniques, and reviewed recruitment materials, consent forms, and the plain English summary. Group composition varied at each meeting, including people with dementia, current and former carers, and people interested in research. Four meetings were held before, and during, data collection.

### Data collection

Before interviews, a semi-structured topic guide was developed (see Supplementary Information S1). The interview guide covered the following domains: a) key people supporting the person with dementia; b) experiences of primary care to date; and c) change ideas. A doctoral researcher (the first author), an academic GP, obtained oral or written consent for interviews and conducted these over the telephone or on Microsoft Teams.

The semi-structured interviews integrated guidance on including people with dementia in qualitative studies and feedback from the PPIE group into their design.^32^ This included regular pauses, allowing the person with dementia to elaborate on points even if these seemed tangential, and building rapport to encourage confidence to speak freely. Interview duration ranged from 38 to 75 minutes. Interviews were audio-recorded and transcribed by a professional transcription company.

### Data analysis

Three researchers contributed to the qualitative analysis (the first author, second author, and senior author). NVivo (version 12) was used to examine all transcripts, code, annotate, refine, and cross-reference themes. Thematic analysis was conducted according to Braun and Clarke’s reflexive thematic analysis framework.^33^ Two researchers (the first author and senior author) examined transcripts independently and familiarised themselves with the data; an initial codebook was developed, with each coder developing themes within the transcripts, subsequently discussed at data meetings. A memorandum was created to summarise each interview individually. This included details about the interview context, initial codes, preliminary themes, and relevant extracts. This was reviewed by all three researchers involved in the analysis. Generated themes were discussed in weekly data meetings where codes were refined, and key themes across interviews discussed, defined, and named. Further data meetings with a third researcher (the second author) were completed, where themes were refined further, definitions of themes were established, and relevant extract examples were selected to underpin points and relate the analysis back to the research question and existing literature.

Reflexivity, the process of the qualitative researcher continually reflecting on their own biases and experiences, and how this influences the research process and findings, was considered throughout data collection and analysis. The researchers’ roles and influence on the data was considered as described in the literature exploring reflexivity in qualitative research,^34,35^ and Braun and Clarke’s reflexive thematic analysis framework.^33–36^ The interviewer kept a reflexive diary, reflecting on her own influence on the data generated, particularly in the context of her professional role as a GP, interviewing people about primary care.^34^ Data collection was deemed complete when no new themes were being generated and new data were repeating concepts expressed in previous data.^37^

## Results

In total, 20 people were interviewed (Table 1). These comprised six dyad interviews, covering 12 participants, six with dementia and six carers; four interviews were with people with dementia alone; and four with carers. Of the participants, four (20%) were first-/second-generation immigrants, two (10%) spoke English as a second language, one (5%) had a disability, and two (10%) were from minority ethnic groups. Ten participants had dementia, with all main subtypes represented. Among the dyad interviews, there was one parent–child, four spousal, and one grandparent–grandchild relationship. All four carers interviewed alone cared for parents with dementia; of these, two had parents in a care home. All people with dementia interviewed lived in their own home, three lived alone. Only one participant was not currently living in an IMD 1/2 quintile postcode according to their JDR database record.

**Table 1.** Basic demographics of the sample

Participant	Gender	Age category, years	Role^a^	IMD quintile
A001	Male	70–79	Person with dementia	1
A002	Male	70–79	Person with dementia	1
A003	Female	40–49	Carer	2
A004	Female	≥80	Person with dementia	2
A005	Male	40–49	Carer	2
A006	Female	60–69	Person with dementia	1
A007	Male	60–69	Carer	1
A008	Female	60–69	Person with dementia	1
A009	Female	60–69	Carer	1
A010	Female	60–69	Person with dementia	2
A011	Male	60–69	Carer	2
A012	Female	30–39	Carer	1
A013	Female	30–39	Carer	3^b^
A014	Female	30–39	Carer	2
A015	Female	70–79	Person with dementia	2
A016	Male	≥80	Person with dementia	1
A017	Male	≥80	Person with dementia	2
A018	Female	≥80	Carer	2
A019	Male	60–69	Carer	2
A020	Female	70–80	Person with dementia	2

^a^Breakdown of dementia subtypes: Alzheimer’s disease, *n *= 5; vascular dementia, *n* = 1; Lewy-body dementia, *n *= 3; and frontotemporal dementia, *n *= 1. ^b^Eligible based on other characteristics despite living in IMD quintile 3 postcode. IMD = Index of Multiple Deprivation.

We identified four key, interrelated themes (Figure 1):

- proactive continuity as a means of maintaining identity amid impending loss;
- the paradox of healthcare support falling away as it becomes needed more;
- challenges with accessing and navigating the primary healthcare system; and
- uncertainty around where dementia health care sits within primary care.

**Figure 1.**
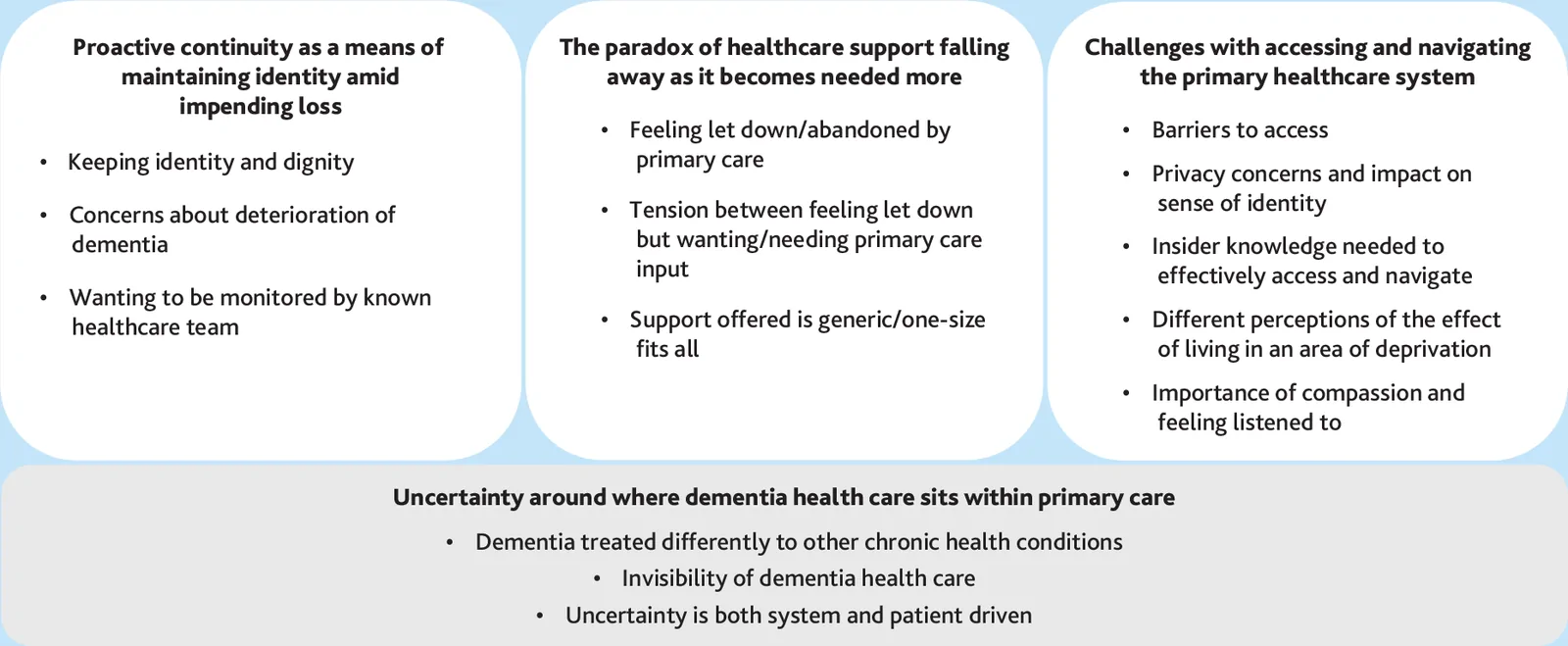
Interrelation of main themes and sub-themes.

The generated themes can be seen as general in terms of living with dementia; we discuss how each of these manifests within the primary care setting or interactions. Supplementary Box S1 provides further illustrative quotes.

### Theme one: proactive continuity as a means of maintaining identity amid impending loss

Participants described a strong sense of identity; wanting to retain this featured across accounts. Participants described different ways in which they (or their loved one) attempted to retain identity, including getting a tattoo to raise awareness of their condition and engaging with dementia organisations. This theme aligned with primary care, with descriptions of participants wanting to remain involved in conversations about their health care, feel valued and listened to, and expressing a desire to be proactively monitored for cognitive deterioration, with a known healthcare worker giving external insight:

*‘I tell everybody I’ve got Alzheimer’s, I talk about it. And I’ve actually had a tattoo on my arm, a forget-me-not tattoo on my arm. It’s a matter of fetching awareness.’* (A001, person with dementia)

*‘When I went to see the lady doctor, she actually talked to me, she didn’t talk to* [A007]*. She didn’t ignore* [A007]*, but she actually turned her chair, and talked to me, and let me answer. That was important. And that makes me feel more of a person.’* (A006, person with dementia)

Some participants embraced their disease identity, with one even naming his Alzheimer’s disease ‘Ali’, and others describing gradual acceptance. Accepting the disease identity seemed to bridge maintenance of prior identity and impending loss:

*‘So I’ve had lots of opportunities and lots of fun, and Ali gets in the way now, with dates and times and remembering to phone people and do things.’* (A016, person with dementia)

People’s sense of identity, both prediagnosis and as a person with dementia, was juxtaposed with understanding of impending loss and that dementia will progress over time. This was underpinned by concerns about loss of insight, and how participants would know if their or their loved one’s dementia had worsened:

*‘I nursed them* [people with dementia] *for 37 years, so I know what it’s like. I know exactly what it’s like and what happens … I know what’s coming.’* (A015, person with dementia)

*‘When you’re thinking about these things, really you need somebody to talk to and one of the … what I just thought about then, was, will I know when I get bad?’* (A001, person with dementia)

Participants valued proactive contact and more time during consultations. There was a sense of needing certainty about where they were in terms of dementia progression, in the context of a longitudinal relationship with their GP. This desire for continuity echoed the desire to be held by, and known to, the primary care team. This was contextualised as nostalgia about how primary care used to be, with doctors and patients known to each other:

*‘*[About seeing a GP regularly] *you'd know where you are, you know, whether things are improving, whether you're getting any better, or worse, or if we got some information. Whereas at the moment, she’s taking tablets just for the sake of taking them.’* (A007, spouse of person with dementia)

*‘I hark back to when I used to go to the same doctor all the time. They knew you, they knew your family, they knew your brothers and sisters, they knew your whole history. And it was so much better … You don’t have to go over everything again and again and again, you know, the personal side of a practice is ... it’s crucial, I think.’* (A009, daughter of person with dementia)

### Theme two: the paradox of healthcare support falling away as it becomes needed more

Participants desired proactive monitoring in primary care for certainty amid cognitive decline (theme 1). Paradoxically, healthcare support was seen to reduce post-diagnosis. Patients struggled to get referrals for diagnosis, then received brief memory team input, then felt abandoned again after this. As specialist support dropped away, primary care became the sole perceived source of ongoing help:

*‘... that was probably April, May time maybe of 2020. She didn’t actually get a diagnosis until March of 2021, for a variety of reasons I think. Quite dismissive of the GP. We really had to push for it.’* (A013, daughter of person with dementia)

**A006:** *‘Well they discharged me from the memory clinic, didn’t they?’* (Person with dementia)

**A007:** *‘Yeah, because they handed it onto the doctors, yeah.’* (Spouse of person with dementia)

*‘We’ve lost our specialist. We haven’t got a neurologist anymore. The neurologist has dropped off, the psychiatrist has dropped off and we’re left with* [specialist centre] *… But we know that we’ve been dropped off by the memory clinic now. They’ve been putting her on some tablets … so she’s on those now … they’ve dropped off the radar.’* (A019, spouse of person with dementia)

There was a sense that after diagnosis some people were ‘passed back’ to primary care. This aligns with the current UK context of referral to diagnostic services without ongoing specialist follow-up:

*‘Because* [place name] *walk-in centre, you know, that part that dealt with the initial diagnosis, and gave us some medication, they just seem to have handed it all onto the doctors, and the doctors haven't done anything at all.’* (A007, spouse of person with dementia)

Participants largely found primary care for people with dementia inadequate. Even those offered post-diagnostic healthcare support felt it was generic, overwhelming, or poorly timed, leading to feelings of abandonment. A gap existed between the primary care provided and what participants needed. This aligned with theme one, where people desired proactive continuity to maintain their identity through a connection with a known healthcare provider:

*‘It was maybe a little bit mindboggling, all the different pockets of support that were available.’* (A013, daughter of person with dementia)

*‘... when* [A020] *was ill the GPs wouldn’t even come out to see her. They said no, we can only go and see people who are, is it bedridden, or whatever. I said,* [A020] *can’t even get out of bed, she was so bad. And in the end* [doctor] *came; I think he felt guilty. I sent a letter. I was one step off finding where I could send an official complaint and sending an official complaint to them ... You see, we want to keep on the side with the GPs, we don’t want to fall out with them, we need them.’* (A019, spouse of person with dementia)

### Theme three: challenges with accessing and navigating the primary healthcare system

Participants found primary health care challenging to access and navigate, describing issues with getting appointments, privacy, having enough time, and complexity. This supported the concepts described in themes one and two, wanting to maintain identity and dignity, and remain known to the primary care team, but healthcare support becoming more difficult to access as dementia progressed:

*‘Yeah, I really struggle, because I’m the one who normally makes the appointment, and I dread calling because you’re that long in a queue waiting. Sometimes you’re waiting that long and then the phone just drops, and then you’ve got to get back in the queue again.’* (A014, granddaughter of person with dementia)

*‘*... *well when we got to reception, and* [male name] *went to book my name in, she asked him why I couldn’t come, and book in. And he told her that I’d got dementia, and that really annoyed me, because it was in front of everybody.’* (A006, person with dementia)

Participants described, through prior experience or knowledge (for example, healthcare roles and caregiving), learning to navigate and access the healthcare system more effectively. This insider knowledge was seen as crucial to access healthcare support needed:

*‘And I do question if I wasn’t a trained healthcare professional who knew the inside workings of services, I don’t think we’d have had as much in the way of help. Because I wonder how people who don’t know, know what to ask, do you know what I mean?’* (A012, daughter of person with dementia)

*‘Yeah. I’ll tell you what it is, until you meet it you have no knowledge of it, and you find little bits of knowledge are given to you.’* (A019, spouse of person with dementia)

Views differed about the impact of living in a socioeconomically disadvantaged area on health care. Some participants felt it made no difference. Others felt the area did not make a difference, but that their individual support network was central to ensuring successful navigation. Participants described charities, local organisations, and family as crucial informal support networks, but did not feel these were impacted by living in a socioeconomically deprived area:

*‘I think even if I had a lot of money, I wouldn’t get a lot more care, because I don’t think there is a lot more care.’* (A001, person with dementia)

*‘I live in* [place name] *... It’s a very deprived town, but I don’t feel like it is, that’s ... it’s hard to describe … It certainly has its challenges but, I mean, thinking about my dad, in particular now, and as well as the rest of my family which is very large. You know, we still are able to take my dad out to visit a local park, take him out and get him some fresh air on the promenade at the seaside. It’s nice* [place] *to live in lots of ways, you know, there’s lots of difficulties for people, there’s an undercurrent in* [place name] *but, you know, it can be a really nice place.’* (A009, daughter of person with dementia)

Participants described frustrations with primary care, including basic questions about their dementia remaining unanswered, GPs being unaware of the diagnosis, and being forced to turn to other sources for support. In contrast, positive experiences with health care centred around being known and listened to:

**Interviewer:** *‘So if you did have a medical question about vascular dementia or you wanted some input about that how would you go about getting some advice?’*

**A014:** *‘We just normally look on Google, don’t we? ... I know it’s not the right place in a lot of senses but if it’s not a really serious question we tend to Google quite a few things. But other than that the GP’s not even mentioned it, have they? ... They’ve not said oh, how are you getting on with your dementia? It’s like they’re not aware that she’s got it.’* (Granddaughter of person with dementia)

*‘They* [the Admiral nurse] *came down to see me. And if I remember rightly, when I had a changeover from one nurse to another, she fetched the other nurse down to introduce her. So, when I ring her, I have an idea of who I’m talking to, if you like. I cannot fault the treatment I have off the Alzheimer nurses, no.’* (A001, person with dementia)

Overall, the sense from participants was needs were not being met by primary care regardless of area, and living in an area of deprivation presented additional challenges:

*‘So my mum lived in the* [place]*, which is a really deprived area, and I live in a more affluent area, and I see the difference from having been in both practices ... my practice seems more proactive, listening, they really promote the NHS app … I can look at my medications, I can look at my blood results, I can analyse them, I can think about my own health and wellbeing.’* (A003, daughter of person with dementia)

### Theme four: uncertainty around where dementia health care sits within primary care

Participants described feeling dementia was treated differently to other chronic health conditions. This was compounded by the sense that participants recognised they were being called proactively for reviews of their other health conditions, but not for dementia:

*‘I think it’s a bit like, if you have high cholesterol, if you have diabetes, you have all of these services offered to you, I think dementia should have that same setup.’* (A003, daughter of person with dementia)

*‘The doctor that I know does do an annual check-up, round about Christmas time, I get wheeled in for a full MOT, as he calls it and we do talk about things but not specifically about that* [dementia] *diagnosis.’* (A002, person with dementia)

Dementia reviews were not clearly identified as such by primary care, meaning participants did not recognise these had happened. Dementia-related health care was taking place, but it was not being communicated clearly to patients, making it invisible to them. Medication reviews and physical health monitoring may have been part of holistic care for people with dementia, but they were not explicitly described as dementia-related primary care:

*‘After 12 months a lady did come to see* [A017] *… she was connected to our doctor. She went through a questionnaire asking. I mean, I was answering the questions really, just asking in general how he was, you know. But I couldn’t say much because he was very, very, good, he hadn’t really changed at all.’* (A018, spouse of person with dementia)

Some participants felt frustrated at how service providers seemed to pick-and-choose which health conditions they engaged with, with concerns about memory being neglected. There was uncertainty around what GPs know about dementia:

*‘You've got to tell the ‘doctor receptionist’ everything. And yet, any time they want you, like, they phoned up a couple of weeks ago, and they said, we need to make an appointment for* [female name]*, because she needs a cardiovascular review. So, whenever they want to get hold of you, it’s got to be on their terms. Whereas, when you phone them, it’s on their terms, you've got to speak to the receptionist, and tell them what you're after.’* (A007, spouse of person with dementia)

*‘I haven't spoken to as many GPs as I have other clinicians. It is maybe down to that. I am unsure ... really, I am unsure what they know.’* (A008, person with dementia)

Uncertainty stemmed from both the primary healthcare system and patients. The primary healthcare system’s proactive dementia care differed from that offered for other conditions. Patients uncertain about primary care team’s dementia knowledge or what could be done were less likely to book appointments for concerns about memory than for physical health issues:

*‘We've had appointments about other stuff. I think the anxiety or depression or whatever is causing her to have a faster heartbeat or experiencing breathlessness so we were contacting the GP about those.’* (A005, son of person with dementia)

This suggests miscommunication may exist between patients and primary care clinicians regarding how dementia should be discussed. This concept links back to theme one, where participants embraced their disease identity as someone with dementia, wanting to remain ‘known’ within a healthcare context.

## Discussion

### Summary

Four main themes were identified. These were: proactive continuity as a means of maintaining identity amid impending loss; the paradox of support falling away as it becomes needed more; challenges with accessing and navigating the primary healthcare system; and, underpinning these three themes, uncertainty around where dementia health care sits within primary care.

### Strengths and limitations

To our knowledge, this is the first study to purposively sample people with dementia and their carers living in socioeconomically disadvantaged areas exploring perspectives on their current experience of primary care. Eight participants were from the poorest 20% of UK neighbourhoods, a group rarely included in research.^38^

There are limitations. Participants were aware of the researcher’s identity as a GP; this may have influenced openness. Despite this, most participants did seem to speak freely, recognising the interviewer’s role, but disclosing negative experiences anyway. Furthermore, the researcher’s identity was considered reflexively in analyses of the generated data by the research team.^34^

As IMD is an area-level measure, individual characteristics may have classified participants’ socioeconomic status differently. Despite this, even if people had higher (or lower) socioeconomic status than their postcode suggests, they still lived in an area of relative deprivation; insights in this context are valuable. It is unclear to what extent our findings are reflective of primary care for people with dementia generally, and to what extent these are experiences solely in areas of socioeconomic deprivation. To examine this further, we would need to conduct a study in high-income areas or use comparison groups.

### Comparison with existing literature

This study expands on existing primary healthcare literature for people with dementia by including perspectives from people living in socioeconomically disadvantaged areas. Although many studies focus on diagnosis and clinician viewpoints,^39–43^ this study emphasised patient and caregiver perspectives about post-diagnostic health care. Our findings align with previous studies examining experiences of primary care for people with dementia. Themes of identity,^14,44^ invisibility of primary care,^20^ and difficulties with navigating the system (‘logistics’)^20^ have been described. Our data are perhaps reflective of experiences of primary care for people with dementia generally, not just in the context of socioeconomically deprived areas.

Participants prioritised maintaining identity, which has been described previously.^44^ This manifested as wanting ongoing monitoring for deterioration by known primary care teams, aligning with the holding concept described in social prescribing literature.^45^ Primary care often failed to provide this proactive support, a problem compounded by system navigation difficulties. These findings support the recently proposed ‘care ecosystem’ model for dementia,^12,46,47^ where proactive support from a dementia navigator was shown to improve outcomes. They also fit with work exploring continuity,^48^ and suggest the idea of a ‘named GP’ or responsible clinician may be valuable to those in lower-income areas.

Previous studies indicate primary care clinicians lack knowledge and confidence in managing dementia.^5,15–17,49^ However, they also feel well suited to provide dementia care because of existing, longitudinal patient relationships and compassion.^15^ Our study suggests this is a dual perspective from those receiving health care and those providing it, with participants describing a tension between wanting more health care from primary care teams, valuing compassionate encounters, but feeling this was not provided in the current system. This indicates an untapped potential for primary care in post-diagnostic dementia health care. Addressing the uncertainty around dementia’s place in primary care is crucial, especially as guidance suggests a shift towards primary care-led dementia management.^8^ Our findings highlight that patients perceive dementia is treated differently to other chronic conditions in primary care, a view previously described only in healthcare workers, to our knowledge.^41^ Clinicians report discussing dementia diagnosis is challenging.^50,51^ This may extend to concerns about discussing dementia generally. Our data suggest people with dementia actively want their dementia recognised, and feel its different treatment represents a gap in primary care.

Our findings align with previous research on inequalities in primary care commissioning for dementia services,^13,30^ which found geographical differences in commissioned support, leading to service gaps, and inequalities in health care provided based on varied intersectional characteristics. We found diverse views on how deprivation and social capital have an impact on healthcare experiences. A new finding, to our knowledge, is that insider, healthcare system knowledge was perceived as important for navigating health care.

### Implications for research and practice

Participants wanted proactive health care from a known primary care clinician, supporting them to understand how their dementia was progressing. Changes within the remit of primary care clinicians’ skills could improve people’s experiences. These include communicating clearly when dementia-related health care is taking place, in the same way this happens for other chronic conditions. Participants wanted to maintain their identity; clinicians could support this through ensuring privacy in accessing health care, actively listening to the person with dementia and their carer, and giving more time. A named clinician, taking responsibility for a person with dementia, as is already suggested in guidance, may bring benefits. Linking back to ongoing work establishing models of best practice, the role of dementia navigators should be explored. These are trained workers who understand the complexity of the community-based landscape of services and provide person-centred support.^12,46,47^ These actions may help to reduce the uncertainty around where dementia sits within primary care for both patients and clinicians, including around transitions after diagnostic assessment. This may include more formal, communicated transfers of health care to primary care, rather than the current paradoxical perceived falling away of support as needs increased.

Further research needs to determine if identified themes are generalisable to people with dementia from all socioeconomic strata, or if they are reflective of living in areas of socioeconomic disadvantage.Future studies, perhaps with comparison groups from diverse socioeconomic backgrounds, may provide more understanding. This would support integration of these important perspectives into emerging models of primary care for people with dementia.

## Data

Data for this study are not publicly available.

## References

[1] Nichols E, Vos T (2021). The estimation of the global prevalence of dementia from 1990‐2019 and forecasted prevalence through 2050: an analysis for the Global Burden of Disease (GBD) study 2019. Alzheimers Dementia.

[2] Alzheimer’s Research UK Dementia statistics hub. https://www.dementiastatistics.org.

[3] Wittenberg R, Hu B, Jagger C (2020). Projections of care for older people with dementia in England: 2015 to 2040. Age Ageing.

[4] Yang M, Chang C-H, Carmichael D (2016). Who is providing the predominant care for older adults with dementia?. J Am Med Dir Assoc.

[5] Sideman AB, Jesus A, Brooks-Smith-Lowe S (2025). Family physicians’ perspectives on outcomes, processes, and policies in dementia care. Health Aff Sch.

[6] National Institute for Health and Care Excellence (NICE) (2018). Dementia: assessment, management and support for people living with dementia and their carers. NG97. https://www.nice.org.uk/guidance/ng97.

[7] All-Party Parliamentary Group on Dementia (2023). Raising the barriers: an action plan to tackle regional variation in dementia diagnosis in England. https://www.alzheimers.org.uk/sites/default/files/2023-10/Raising%20the%20Barriers.pdf.

[8] Gauthier S, Webster C, Servaes S (2022). World Alzheimer Report 2022. Life after diagnosis: navigating treatment, care and support. https://www.alzint.org/u/World-Alzheimer-Report-2022.pdf.

[9] American Academy of Family Physicians Primary care. https://www.aafp.org/about/policies/all/primary-care.html.

[10] Bamford C, Poole M, Brittain K (2014). Understanding the challenges to implementing case management for people with dementia in primary care in England: a qualitative study using Normalization Process Theory. BMC Health Serv Res.

[11] Frost R, Walters K, Aw S (2020). Effectiveness of different post-diagnostic dementia care models delivered by primary care: a systematic review. Br J Gen Pract.

[12] Possin KL, Merrilees JJ, Dulaney S (2019). Effect of collaborative dementia care via telephone and internet on quality of life, caregiver well-being, and health care use: the care ecosystem randomized clinical trial. JAMA Intern Med.

[13] Frost R, Walters K, Wilcock J (2020). Mapping post-diagnostic dementia care in England: an e-survey. JICA.

[14] Bamford C, Wheatley A, Brunskill G (2021). Key components of post-diagnostic support for people with dementia and their carers: a qualitative study. PLoS One.

[15] Sideman AB, Ma M, Hernandez de Jesus A (2023). Primary care practitioner perspectives on the role of primary care in dementia diagnosis and care. JAMA Netw Open.

[16] Foley T, Boyle S, Jennings A, Smithson WH (2017). “We’re certainly not in our comfort zone”: a qualitative study of gps’ dementia-care educational needs. BMC Fam Pract.

[17] Jennings AA, Foley T, McHugh S (2018). “Working away in that grey area…” a qualitative exploration of the challenges general practitioners experience when managing behavioural and psychological symptoms of dementia. Age Ageing.

[18] NICE (2025). Dementia: scenario: follow up of confirmed dementia in primary care. https://cks.nice.org.uk/topics/dementia/management/follow-up-of-confirmed-dementia-in-primary-care/.

[19] NHS England (2023). Quality and Outcomes Framework, 2022–23. https://digital.nhs.uk/data-and-information/publications/statistical/quality-and-outcomes-framework-achievement-prevalence-and-exceptions-data/2022-23.

[20] Wheatley A, Brunskill G, Dow J (2022). The primary care annual dementia review: a qualitative study of the views and experiences of service users and providers. medRxiv.

[21] Peterson K, Hahn H, Lee AJ (2016). In the information age, do dementia caregivers get the information they need? Semi-structured interviews to determine informal caregivers’ education needs, barriers, and preferences. BMC Geriatr.

[22] Morris C, Techache R, Davies K (2024). Inequalities in the quality and safety of post-diagnostic primary care for people with dementia: a scoping review. Int J Geriatr Psychiatry.

[23] Cooper C, Blanchard M, Selwood A, Livingston G (2010). Antidementia drugs: prescription by level of cognitive impairment or by socio-economic group?. Aging Ment Health.

[24] Cooper C, Lodwick R, Walters K (2016). Observational cohort study: deprivation and access to anti-dementia drugs in the UK. Age Ageing.

[25] Mercer SW, Zhou Y, Humphris GM (2018). Multimorbidity and socioeconomic deprivation in primary care consultations. Ann Fam Med.

[26] Asaria M, Cookson R, Fleetcroft R, Ali S (2016). Unequal socioeconomic distribution of the primary care workforce: whole-population small area longitudinal study. BMJ Open.

[27] O’Brien BC, Harris IB, Beckman TJ (2014). Standards for reporting qualitative research: a synthesis of recommendations. Acad Med.

[28] Appel C (2024). Understanding the Index of Multiple Deprivation (IMD) in public health research. https://www.heec.co.uk/resource/understanding-the-index-of-multiple-deprivation-imd-in-public-health-research.

[29] Department for Communities and Local Government (2015). The English Index of Multiple Deprivation (IMD) 2015 – guidance. https://assets.publishing.service.gov.uk/media/5a7f0e5ded915d74e33f410b/English_Index_of_Multiple_Deprivation_2015_-_Guidance.pdf.

[30] Giebel C (2024). A new model to understand the complexity of inequalities in dementia. Int J Equity Health.

[31] Ministry of Housing, Communities and Local Government English indices of deprivation 2019. https://imd-by-postcode.opendatacommunities.org/imd/2019.

[32] Samsi K, Manthorpe J (2020). Interviewing people living with dementia in social care research: methods review. https://sscr.nihr.ac.uk/wp-content/uploads/2025/04/M022-SSCR-methods-review_MR022.pdf.

[33] Braun V, Clarke V (2006). Using thematic analysis in psychology. Qual Res Psychol.

[34] Chew-Graham CA, May CR, Perry MS (2002). Qualitative research and the problem of judgement: lessons from interviewing fellow professionals. Fam Pract.

[35] Olmos-Vega FM, Stalmeijer RE, Varpio L, Kahlke R (2023). A practical guide to reflexivity in qualitative research: AMEE guide no. 149. Med Teach.

[36] Jamieson MK, Govaart GH, Pownall M (2023). Reflexivity in quantitative research: a rationale and beginner’s guide. Social Personality Psych Compass.

[37] Saunders B, Sim J, Kingstone T (2018). Saturation in qualitative research: exploring its conceptualization and operationalization. Qual Quant.

[38] National Institute for Health and Care Research (2024). Improving inclusion of under-served groups in clinical research: guidance from INCLUDE project. https://www.nihr.ac.uk/improving-inclusion-under-served-groups-clinical-research-guidance-include-project.

[39] Milne AJ, Woolford HH, Mason J, Hatzidimitriadou E (2000). Early diagnosis of dementia by GPs: an exploratory study of attitudes. Aging Ment Health.

[40] Koch T, Iliffe S, EVIDEM-ED project (2010). Rapid appraisal of barriers to the diagnosis and management of patients with dementia in primary care: a systematic review. BMC Fam Pract.

[41] Harris DP, Chodosh J, Vassar SD (2009). Primary care providers’ views of challenges and rewards of dementia care relative to other conditions. J Am Geriatr Soc.

[42] Boise L, Camicioli R, Morgan DL (1999). Diagnosing dementia: perspectives of primary care physicians. Gerontologist.

[43] Bernstein A, Rogers KM, Possin KL (2019). Primary care provider attitudes and practices evaluating and managing patients with neurocognitive disorders. J Gen Intern Med.

[44] Telenius EW, Eriksen S, Rokstad AMM (2020). I need to be who I am: a qualitative interview study exploring the needs of people with dementia in Norway. BMJ Open.

[45] Westlake D, Wong G, Markham S (2024). "She’s been a rock”: the function and importance of “holding" by social prescribing link workers in primary care in England — findings from a realist evaluation. Health Soc Care Community.

[46] Sideman AB, Dulaney S, Merrilees J (2025). Dissemination of the care ecosystem collaborative care model for dementia. J Am Geriatr Soc.

[47] Guterman EL, Kiekhofer RE, Wood AJ (2023). Care ecosystem collaborative model and health care costs in medicare beneficiaries with dementia: a secondary analysis of a randomized clinical trial. JAMA Intern Med.

[48] Delgado J, Evans PH, Gray DP (2022). Continuity of GP care for patients with dementia: impact on prescribing and the health of patients. Br J Gen Pract.

[49] Bourque M, Foley T (2020). Improving the quality of dementia care in general practice: a qualitative study. Front Med.

[50] Živanović S, Pavlović J, Hadživuković N (2025). Healthcare workers’ attitudes regarding the assessment of dementia in the elderly. BMC Health Serv Res.

[51] Yates J, Stanyon M, Samra R, Clare L (2021). Challenges in disclosing and receiving a diagnosis of dementia: a systematic review of practice from the perspectives of people with dementia, carers, and healthcare professionals. Int Psychogeriatr.

